# The *Boechera* Genus as a Resource for Apomixis Research

**DOI:** 10.3389/fpls.2019.00392

**Published:** 2019-04-02

**Authors:** Vladimir Brukhin, Jaroslaw V. Osadtchiy, Ana Marcela Florez-Rueda, Dmitry Smetanin, Evgeny Bakin, Margarida Sofia Nobre, Ueli Grossniklaus

**Affiliations:** ^1^Theodosius Dobzhansky Center for Genome Bioinformatics, St. Petersburg State University, Saint Petersburg, Russia; ^2^Department of Plant Embryology and Reproductive Biology, Komarov Botanical Institute RAS, Saint Petersburg, Russia; ^3^Department of Plant and Microbial Biology, Zürich-Basel Plant Science Center, University of Zurich, Zurich, Switzerland; ^4^Bioinformatics Institute, Saint Petersburg, Russia

**Keywords:** genome assembly, *Boechera*, apomixis, apomeiosis, diplospory, pseudogamy, genomics, heterozygosity

## Abstract

The genera *Boechera* (A. Löve et D. Löve) and *Arabidopsis*, the latter containing the model plant *Arabidopsis thaliana*, belong to the same clade within the Brassicaceae family. *Boechera* is the only among the more than 370 genera in the Brassicaceae where apomixis is well documented. Apomixis refers to the asexual reproduction through seed, and a better understanding of the underlying mechanisms has great potential for applications in agriculture. The *Boechera* genus currently includes 110 species (of which 38 are reported to be triploid and thus apomictic), which are distributed mostly in the North America. The apomictic lineages of *Boechera* occur at both the diploid and triploid level and show signs of a hybridogenic origin, resulting in a modification of their chromosome structure, as reflected by alloploidy, aneuploidy, substitutions of homeologous chromosomes, and the presence of aberrant chromosomes. In this review, we discuss the advantages of the *Boechera* genus to study apomixis, consider its modes of reproduction as well as the inheritance and possible mechanisms controlling apomixis. We also consider population genetic aspects and a possible role of hybridization at the origin of apomixis in *Boechera.* The molecular tools available to study *Boechera*, such as transformation techniques, laser capture microdissection, analysis of transcriptomes etc. are also discussed. We survey available genome assemblies of *Boechera* spp. and point out the challenges to assemble the highly heterozygous genomes of apomictic species. Due to these challenges, we argue for the application of an alternative reference-free method for the comparative analysis of such genomes, provide an overview of genomic sequencing data in the genus *Boechera* suitable for such analysis, and provide examples of its application.

## Gametophytic Apomixis and Its Relevance to Agriculture

Apomixis is defined as the asexual reproduction through seeds and results in the formation of genetically uniform progeny (Nogler, 1984a; Asker and Jerling, 1992; Grossniklaus, 2001; Bicknell and Koltunow, 2004; Van Dijk, 2009; Kotani et al., 2014). During sexual reproduction, egg and central cell – the gametes of the reduced female gametophyte (embryo sac) – each get fertilized by one sperm cell to produce the embryo and endosperm, respectively (Dresselhaus et al., 2016). In contrast, apomictic embryos are not the result of a fusion of male and female gametes but develop clonally from unreduced maternal cell lineages in the ovule (matroclinous inheritance). Characteristic components of gametophytic apomixis are (i) avoidance of meiosis (apomeiosis), (ii) development of the embryo from an unreduced egg cell without fertilization (parthenogenesis), and (iii) formation of functional endosperm either autonomously or by fertilization of the central cell (pseudogamy) (Koltunow, 1993; Grossniklaus, 2001; Koltunow and Grossniklaus, 2003; Hand and Koltunow, 2014).

A drawback of sexual propagation is the segregation of advantageous traits in subsequent generations, such that progeny can lose the advantageous gene combinations of their parents (Spillane et al., 2004; Brukhin, 2017). The study of apomixis has drawn greater interest over the last two decades because of its potential to fix agriculturally valuable characteristics over many generations. The introduction of apomixis into crop plants would allow the long-term fixation of complex genotypes, including those of F1 hybrids often used in agriculture. This would facilitate crop breeding and hybrid seed production and could greatly benefit subsistence farmers by providing them access to high-yielding hybrid crops (Grossniklaus et al., 1998; Spillane et al., 2004; Conner and Ozias-Akins, 2017). Many possible uses of apomixis in agriculture have been proposed and its importance for sustainability and food security has been recognized (Karpechenko, 1935; Jefferson, 1994; Toennissen, 2001; Grossniklaus et al., 2003; Spillane et al., 2004; Conner and Ozias-Akins, 2017). Unfortunately, almost no natural gametophytic apomicts have been found among major crop cultivars and the introgression of apomixis from wild apomictic relatives has so far been unsuccessful (Savidan, 2000).

As an alternative to introgression, genes relevant to apomixis could either be identified in sexual model systems by identifying mutants displaying components of apomixis, or by isolating the relevant genes from an apomictic species (e.g., Grossniklaus, 2001; Pupilli and Barcaccia, 2012; Rodriguez-Leal and Vielle-Calzada, 2012; Barcaccia and Albertini, 2013; Conner and Ozias-Akins, 2017). For the latter approach, an important question is how to choose a convenient apomictic model plant, which will allow the deciphering of the molecular mechanisms underlying the components of apomixis. Three apomictic genera that have been studied in depth, *Hieracium*, *Paspalum* and *Pennisetum*, have large genomes and are polyploid, which is true for the vast majority of currently known apomicts (Asker and Jerling, 1992; Carman, 1997; Hojsgaard et al., 2014). Although these features complicate molecular genetic studies, research in these natural apomicts have greatly contributed to progress in the field (reviewed in e.g., Ortiz et al., 2013; Bicknell et al., 2016; Conner and Ozias-Akins, 2017). In contrast to the natural apomicts mentioned above, *Boechera* spp. have a relatively small genome (∼170–230 Mb) and *Boechera* is the only known genus where apomixis is found at the diploid level in the wild (Böcher, 1951; Dobeš et al., 2004b; Sharbel et al., 2005; Voigt-Zielinski et al., 2012). In addition, *Boechera* spp. are close relatives of the model plant *Arabidopsis thaliana*, which is very well studied in terms of molecular genetics and functional gene annotation. However, the genomes of apomictic accessions of *Boechera* are characterized by extremely high heterozygosity, accompanied by alloploidy and aneuploidy that resulted from hybridization events (e.g., Schranz et al., 2005; Koch et al., 2003; Mandáková et al., 2015). This poses challenges to perform a phased assembly and detailed annotation (reviewed in Hirsch and Buell, 2013) of the genomes of apomictic *Boechera* accessions.

In this review, we will present the particularities of phylogeny, reproduction, and genetics of the *Boechera* genus, and discuss strategies for assembly and annotation of the genomes of apomictic *Boechera* accessions.

## Taxonomy and Habitats of the Most Important *Boechera* Species

The genus *Boechera* comprises mainly North American species of biennial and perennial herbaceous crucifers, characterized by a base chromosome number of *n* = 7. Previously, these species were included in the genus *Arabis* L., from which they were excluded based on the difference in the base chromosome number (Löve and Löve, 1976), which is *n* = 8 in *Arabis* spp. Molecular genetic data confirmed the difference between the two genera. It was shown that the similarity between them is convergent, representing two evolutionary independent lineages in Brassicaceae (Al-Shehbaz, 2003). Recently, the taxonomy of the genus *Boechera* has been further developed using molecular markers. Currently, 110 species have been described within the genus, 71 of them are diploid and presumably sexual although diploid apomicts have also been described, and 38 are reported to be apomictic triploids of hybridogenic origin (Windham and Al-Shehbaz, 2006, Windham and Al-Shehbaz, 2007a,b). Thus, *Boechera* is the fifth largest genus within the Brassicaceae.

**FIGURE 1:**
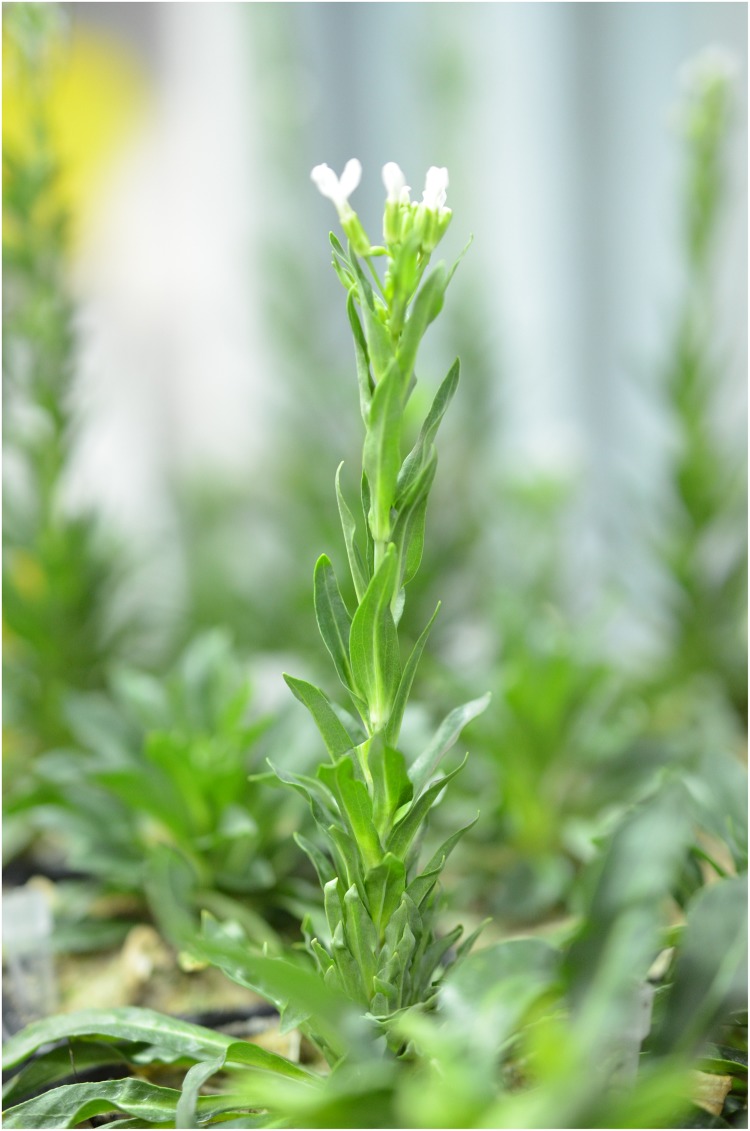
*Boechera stricta* grown in the greenhouse of the Department of Plant and Microbial Biology of the University of Zurich.

Most studies on the reproductive biology of *Boechera* involve just a small number of species. These are the widely distributed sexual diploid *Boechera stricta* (A. Gray) A. Löve & D. Löve (Figure 1), the sexual and apomictic plants previously known under the name *Boechera holboellii* (*sensu lato, s. l.*) (Hornem.) A. Löve & D. Löve, and apomicts of a hybridogenic origin previously referred to as *Boechera divaricarpa* (A. Nelson) A. Löve & D. Löve (Windham and Al-Shehbaz, 2007b). Recently, several studies also used *Boechera gunnisoniana* (Rollins) W.A. Weber (Taşkin et al., 2003, 2004, 2009a; Schmidt et al., 2014; Kirioukhova et al., 2018). The rest of the species were mainly investigated to study particular aspects of apomixis in a geographically large number of species (Aliyu et al., 2010; Corral et al., 2013; Mau et al., 2013, 2015).

Until recently, the Pleistocene relict *B. holboellii* (*Arabis holboellii*) was treated in a broad sense as a species with a scattered range (i.e., consisting of several geographically isolated areas due to reasons of historical nature) (Böcher, 1951). However, recent taxonomic studies using molecular markers showed that it is a polyphyletic, artificial taxon, including a number of distinct species (Windham and Al-Shehbaz, 2006; Alexander et al., 2013). At present, *B. holboellii* is considered in the narrow sense as plants growing in Greenland. It includes sexual and apomictic diploid and triploid forms and the latter ones, unlike the North American species, appear to be autotriploids (Windham and Al-Shehbaz, 2006).

The continental North American accessions, which previously were included in *B. holboellii*, are distinguished as series of individual species that form an agamic complex (Stebbins, 1950). The basis of this complex consists of four diploid sexual species, in which, however, the presence of facultative apomixis cannot be excluded: *Boechera collinsii* (Fernald) A. Löve & D. Löve, *Boechera pendulocarpa* (A. Nelson) Windham & Al-Shehbaz, *Boechera polyantha* (Greene) Windham & Al-Shehbaz, and *Boechera retrofracta* (Graham) A. Löve & D. Löve. The remaining species are triploid apomicts of hybridogenic origin that are morphologically very similar to the parental sexual species: *B. consanguinea* (*retrofracta* × *fendleri*), *B. goodrichii* (*retrofracta* × *gracilipes*), *Boechera grahamii* (*stricta* × *collinsii*), *B. pauciflora* (*sparsiflora* × *retrofracta*), *B. pinetorum* (*rectissima* × *retrofracta* × *sparsiflora*), *Boechera quebecensis* (*holboellii* × *stricta*), and *B. tularensis* (*retrofracta* × *rectissima* × *stricta*) (Windham and Al-Shehbaz, 2007a,b). The *B. quebecensis* is distributed in isolated areas of North-Eastern America, implying the presence of one of its putative parents (Greenlandic *B. holboellii s. s.*) on the North American continent in the past.

The apomictic *B. divaricarpa* is probably the most problematic species in the genus from a taxonomic viewpoint. Traditionally, a large diversity of hybrids involving *B. stricta* as one of the parents (including *B. stricta* × *B. holboellii s. l*.) were referred to as *B. divaricarpa* in many articles on the reproductive biology of the genus *Boechera*. Such uncareful use of the name could be a potential source of confusion. As Windham and Al-Shehbaz (2007b) state, the correct use of the name *B. divaricarpa* should be restricted to plants containing genomes of *B. stricta* and *B. sparsiflora*. For hybrids of *B. stricta* × *B. collinsii*, the name *B. grahamii* should be used. The hybrids of *B. stricta* × *B. holboellii s. s.* should be referred to as *B. quebecensis*. In cases where the second parent of the hybrid is uncertain, the name “*B. divaricarpa*” should be avoided and replaced by “*B. stricta* hybrid.”

In terms of prospective models for the study of apomixis, *B. gunnisoniana* deserves attention. It is a triploid species of presumably hybridogenic origin with diploid sexual species *B. oxylobula* and *B. thompsonii* (=*B. pallidifolia*) as parents (Mateo de Arias, 2015). It is characterized by almost obligate pseudogamous apomixis (Roy, 1995; Taşkin et al., 2004; Schmidt et al., 2014), a small plant size, and relatively fast development (approximately 4 months from planting to seed).

Although the vast majority of species of the genus *Boechera* grows in North America, the occurrence of two putative *Boechera* species in the Russian Far East has been reported, representing an example of East Asian/North American floristic disjunction. *B. falcata* (Turcz.) Al-Shebaz from the Russian Far East is closely related to the well-known North American apomicts (*Boechera s. s.*) based on molecular markers (Al-Shehbaz, 2005; Kiefer et al., 2009; Alexander et al., 2013), and its more detailed study with respect to the potential presence of apomixis is of a great interest. Another species is endemic of the Baikal region and the Russian Far East, *Borodinia* (=*Boechera*?) *macrophylla* (Turcz.) German. Recent molecular genetic studies showed its close relationship with seven *Boechera* species from the Eastern United States (Al-Shehbaz and German, 2010; Alexander et al., 2013).

## Advantages of the *Boechera* Genus for the Study of Apomixis

Over the last decade, various species of the genus *Boechera* have been adopted as a model to study the molecular basis of apomixis, in addition to its well-established role as a study system in evolutionary ecology (reviewed in Rushworth et al., 2011). Among the advantages of *Boechera* spp. as a model are:

(i) Its close relationship to the model plant *A. thaliana* (L.) Heynh. (Huang et al., 2016), for which extensive molecular genetic resources are available, whose genome is fully sequenced and very well annotated, and in which many genes required for reproduction are known, facilitating the search for genes involved in the control of apomixis in *Boechera* spp.;
(ii) The small size of its genome ranging from ∼170–230 Mbp;
(iii) The occurrence of apomixis also at the diploid level (2*n* = 14), representing an exception among apomictic plants (Böcher, 1951; Voigt-Zielinski et al., 2012; Lovell et al., 2017);
(iv) The generation of unreduced spores by diplospory of the *Taraxacum* type (Crane, 2001), which closely resembles sexual, meiotic development but is modified to form two unreduced instead of four reduced spores; different accessions show various levels of synapsis disorders during apomeiosis: fully synaptic, partially synaptic, and completely asynaptic forms exist;
(v) Apomictic *Boechera* spp. being pseudogamous, which is rare among species with diplospory (Talent, 2009);
(vi) Apomixis in the genus *Boechera* always being facultative, allowing for hybridization, and certain genetic analysis even with near-obligate apomictic forms;
(vii) The inclusion of both sexual and apomictic species in the genus *Boechera* with accessions of varied ploidy and geographic origin; the genetic differences between many species is very small, facilitating the search for homologous sequences in molecular studies;
(viii) Sexual *Boechera* accessions being self-compatible and largely self-pollinating, unlike the sexual ancestors of most other apomicts, which are self-incompatible and cross-pollinating; as a consequence of self-pollination, sexual accessions have extremely low heterozygosity;
(ix) The available methods for genetic transformation via somatic embryogenesis in tissue cultures of apomictic *B. gunnisoniana* and *B. holboelliis. l.* (Taşkin et al., 2003, 2009b).

## Cyto-Embryological Studies in the Genus *Boechera*

The first detailed cyto-embryological studies of apomixis in the genus *Boechera* were undertaken by the Danish botanist Tyge W. Böcher (1947, 1951, 1954, 1969). He discovered the presence of apomixis in *B. holboellii s. l.* (referred to by him as *Arabis holboellii*) in diploid and triploid plants, and described megasporogenesis and microsporogenesis in a number of sexual and apomictic *Boechera* accessions from Greenland and North America. Particularly remarkable was his description of forms with varying degrees of chromosome synapsis in meiotic prophase. He also noted the presence of plants with different ploidy levels (mainly 2*n* and 3*n*, rarely 4*n*, 5*n*, and 6*n*) and aneuploids (2*n* = 16, 22, 23, and 30), and assumed a hybrid nature for the latter (Böcher, 1954).

**FIGURE 2:**
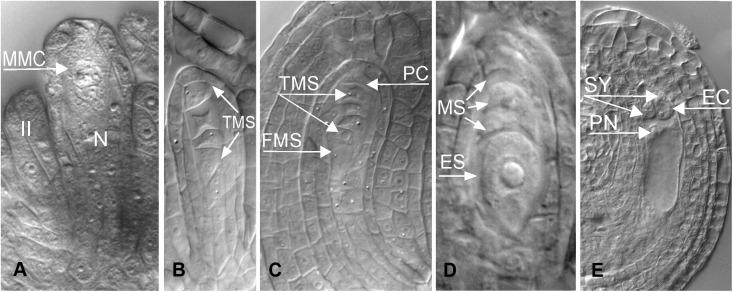
Meiotic (sexual) megasporogenesis in ovules of *B. holboellii s. l.* **(A)** megaspore mother cell (MMC), nucellus (N); **(B**,**C)** tetrad of the megaspores (TMS), the functional megaspore (FMS) at the chalazal end; **(D)** uninucleate meiotic embryo sac (ES) with the remnants of degenerating, non-functional megaspores (MS); **(E)** mature seven-celled *Polygonum* type embryo sac with an egg cell (EC), two synergids (SY), and a central cell with two polar nuclei (PN) **(A**,**C**,**E** – Naumova et al., 2001; **B**,**D** – Osadtchiy et al., 2017).

**FIGURE 3:**
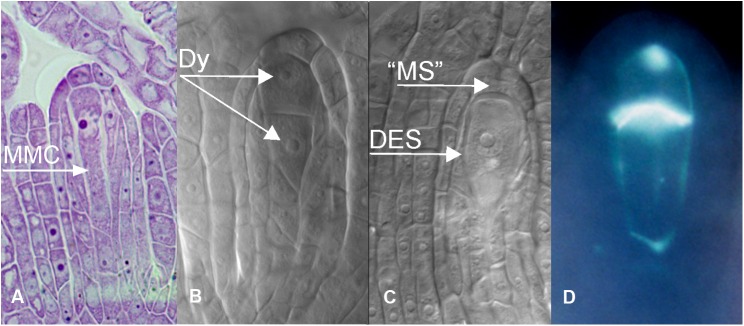
Apomeiotic megasporogenesis in *B. holboellii s. l.* **(A)** megaspore mother cell (MMC); **(B)** diplosporous dyad (Dy); **(C)** uninucleate diplosporous embryo sac (DES) with remnants of the “megaspore” (“MS”); **(D)** callose in the cell wall of a diplosporous dyad **(B**,**C** – Naumova et al., 2001; **A**,**D** – Osadtchiy et al., 2017).

**FIGURE 4:**
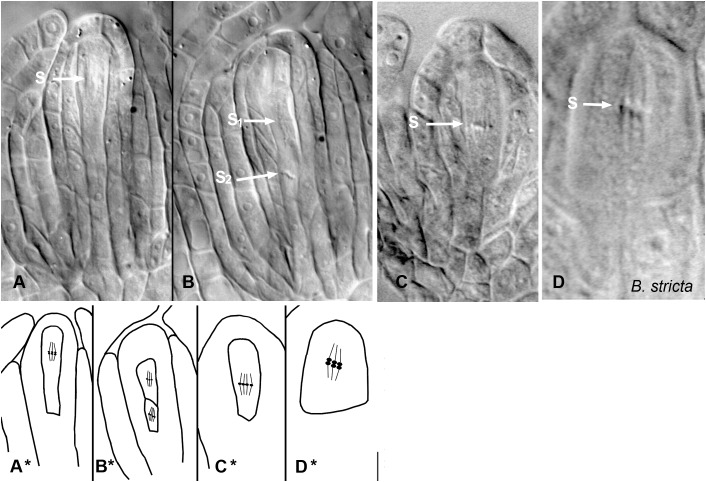
Meiosis and apomeiosis in *B. holboellii s. l*. **(A**–**C**,**A^∗^**–**C^∗^**) and *B. stricta* **(D**,**D^∗^)**. **(A**,**A^∗^)** metaphase I meiotic spindle (S); **(B**,**B^∗^)** two metaphase II meiotic spindles (S1 and S2); **(C**,**C^∗^)** equational division spindle during apomeiosis (S); **(D**,**D^∗^)** metaphase I meiotic spindle (S) in sexual *B. stricta* **(B)** (Osadtchiy et al., 2017).

Nearly 50 years later, a Dutch-Russian team performed embryological studies of cleared specimens of *B. holboellii s. l.* (accessions from Greenland and Colorado) using differential interference contrast microscopy (DIC) together with a flow cytometric seed screen (FCSS) analysis (Naumova et al., 2001). The presence of meiotic and apomeiotic events during megasporogenesis was demonstrated (Figure 2–4). By screening a large number of cleared ovules, the formation of an unreduced embryo sac through diplospory and parthenogenetic development of the embryo in apomictic *Boechera* accessions were confirmed. In sexual accessions, embryo sac development follows the *Polygonum* type (Maheshwari, 1950; Figure 2, 4): the diploid megaspore mother cell (MMC) undergoes meiosis to produce a tetrad of haploid megaspores, three of which degenerate while the functional megaspore undergoes three mitotic divisions to give rise to an eight-nucleate, seven-celled embryo sac comprising an egg cell, two synergids, three antipodal cells, and two polar nuclei that fuse to form the homo-diploid nucleus of the central cell. In the anthers, pollen grains develop from the pollen mother cells (PMCs) that undergo meiosis to produce tetrads of microspores. After their separation, each microspore divides asymmetrically into a large vegetative and a smaller generative cell. After pollination, the vegetative cell germinates producing a pollen tube that transports the sperm cells to the embryo sac, while the generative cell divides once more to form two sperm cells that will later fertilize the egg and central cell, giving rise to a 2*n* embryo and a 3*n* endosperm, respectively (Figure 2, 4).

In apomictic *Boechera* accessions, diplosporous apomeiosis of the *Taraxacum* type (Crane, 2001) is the most common (Böcher, 1951; Naumova et al., 2001; Schmidt et al., 2014; Mateo de Arias, 2015; Windham et al., 2015; Figure 3, 4C). In meiotic diplospory, the embryo sac originates from an MMC that undergoes an aberrant meiosis without chromosome segregation, resulting in the formation of a dyad of unreduced megaspores. A characteristic feature of the MMC is the lack of callose deposition around the cell (Rodkiewicz, 1970; Nogler, 1984a; Carman et al., 1991). In contrast, callose was observed in the cell wall between the two cells of the dyad (Figure 3D). Dyad formation is most commonly observed (Roy, 1995; Taşkin et al., 2004; Schmidt et al., 2014); however, rare triads and even tetrads can be found (Schmidt et al., 2014). The chalazal dyad cell undergoes three rounds of mitosis, producing an unreduced eight-nucleate embryo sac that is morphologically similar to the *Polygonum* type (Crane, 2001; Rojek et al., 2018).

Aposporous apomeiosis was previously thought to be uncommon in *Boechera* spp., but recent reports described its occurrence in several species. The overwhelming majority of aposporous embryo sacs developed according to the *Hieracium* type (Crane, 2001). The sexual MMC in this case might degenerate or undergo meiosis as was observed in rare instances in *B. microphylla* (Carman, 2007; Mateo de Arias, 2015; Carman^1^). In *B. retrofracta* × *stricta* hybrids, diplospory of the *Antennaria* type (Crane, 2001) was also observed rarely (Carman see text footnote 1). In a FCSS, it was also shown that the percentage of mature sexual seeds (in relation to apomictic seeds) in all studied apomicts was significantly lower than the percentage of morphologically normal meiotic tetrads (in relation to apomeiotic events). This implies that meiosis proceeds abnormally in the most cases, resulting in inviable seeds, while apomixis serves as an “escape from sterility” (Mateo de Arias, 2015).

In most apomicts, pollen development is unaffected. In *Boechera* spp., however, apomeiosis also occurs during microsporogenesis (Böcher, 1951; Taşkin et al., 2009a). In triploid apomicts, meiosis I fails as the chromosomes are unable to correctly pair at pachytene. The chromosomes migrate to opposing poles of the PMC and decondense. After cytokinesis the dyad, unlike the meiotic tetrad, is enclosed by a callose wall. Chromosome synapsis with the formation of bi- and trivalents in the metaphase I occurs in *B. holboellii s. l*., whereas apomeiosis in *B. gunnisoniana* is completely asynaptic. Investigation of microsporogenesis in apomictic triploid *B. holboellii s. l.* and *B. gunnisoniana* showed that in the triploids, the majority of pollen grains are unreduced, formed through apomeiotic dyads (98% in *B. holboellii s. l.*, 90% in *B. gunnisoniana*), while the rest of the pollen was formed through (partially abnormal) meiosis, resulting in tetrads or triads of microspores (Taşkin et al., 2009a), or sometimes even in monads in triploid *B. holboellii s. l.* (Böcher, 1951). In diploid apomicts, variability in apomeiosis is higher. In different accessions of the *B. holboellii* complex, pairing and cross-over events can occur normally at pachytene in some accessions, resulting in reduced pollen, or trivalents and even quadrivalents can be formed in others. Some accessions showed mostly diploid pollen formation, while others displayed evidence of haploid and diploid pollen (Kantama et al., 2007; Taşkin et al., 2009a; Kirioukhova et al., 2018). In fact, it has been observed that both diploid and triploid apomicts can produce reduced and unreduced pollen in varying proportions (Böcher, 1951; Voigt et al., 2007; Aliyu et al., 2010; Voigt-Zielinski et al., 2012).

In sexually reproducing *Boechera* spp. double fertilization occurs, while in apomicts only fertilization of the polar nuclei takes place. However, fully autonomous apomixis can also be found in rare cases (Matzk et al., 2000; Naumova et al., 2001), it is more often observed in triploids (at frequencies of up to 15%) than in diploids (1.33% at most) (Aliyu et al., 2010). The formation of embryos with a doubled set of chromosomes as a result of the fusion of unreduced male and female gametes can also occur as a rare event (Naumova et al., 2001). An important conclusion of the FCSS-based study was that, in apomicts, all mature seeds were derived from unreduced female and male gametes (Naumova et al., 2001; Table 1).

**Table 1:** The reproductive modes in *B. holboellii s. l.* accessions (based on cyto-embryological investigation of ovules and FCSS) (from Naumova et al., 2001).

	Female gametophyte development			
Accession	(based on cyto-embryological data)				Seed development (FCSS)	
		
						C values embryo:endosperm
	# plants	# ovules	% of apomeiosis		# plants	(# seeds)
					
			Average	Range		
Colorado triploids	4	671	98	95–100	4	3C:9C(40)^a^ 3C:6C(5)^b^
Greenland diploids	16	462	73	45–89	5	2C:6C(55)^a^ 2C:4C(1)^b^ 4C:6C(1)^c^
Greenland triploids	4	513	94	83–98	2	3C:9C(21)^a^ 3C:6C(1)^b^ 6C:9C(1)^c^


*^*a*^Unreduced embryo sac (ES), parthenogenetic embryo, and pseudogamous endosperm development. ^*b*^Unreduced ES, parthenogenetic embryo, and autonomous endosperm development. ^*c*^Unreduced ES and double fertilization by unreduced pollen.*

In apomicts, endosperm ploidy varies according to the ploidy of the sperm cells, although the most common ratio is a 2 maternal:1paternal (2m:1p) genome ratio. Exceptions, although at lower frequencies, do exist, indicating that there is some degree of flexibility or, at least, that the system is “leaky” (Aliyu et al., 2010).

A large-scale FCSS, covering 16 *Boechera spp*. revealed a wide variability in reproductive mode within diploid genotypes, ranging from obligate sexual to nearly obligate apomictic. By assessment of the percentage of apomeiosis, sexual and parthenogenetic embryo formation, as well as sexual, pseudogamous, and autonomous endosperm development, it was shown that all investigated facultative apomicts of the same genotype had either a very low (1–3%) or a very high (87–99%) percentage of the apomeiosis, and individuals with intermediate frequencies were not observed. Furthermore, all triploids were found to be obligate apomeiotic. A genotype-specific correlation between apomeiosis on the one hand, and parthenogenesis combined with pseudogamous or autonomous endosperm development on the other hand, showed that frequencies of the latter never exceeded the frequency of apomeiosis (Aliyu et al., 2010). This may indicate a close relationship of their genetic control and a key role of apomeiosis for all subsequent stages of apomictic development.

## Population Genetic Studies in the Genus *Boechera* With Respect to Apomixis

Apomictic and sexual lineages within the genus *Boechera* can inter-cross (Schranz et al., 2005; Alexander et al., 2013). Distinct evolutionary forces are expected to drive the evolution of lineages that differ in their reproductive modes. In sexual lineages, recombination increases the probability of elimination of deleterious mutations (Hill-Robertson effect, Hill and Robertson, 1966; Felsenstein, 1976). Apomictic lineages, in contrast, reproduce asexually and do not undergo recombination; thus they cannot recover adaptive alleles once deleterious mutations occur within these alleles (Charlesworth, 2008). Therefore, one expects the accumulation of deleterious alleles, a phenomenon known as Muller’s ratchet (Muller, 1964; Charlesworth and Charlesworth, 1997). Recent comparisons of apomictic and sexual lineages in *Boechera* spp. have supported these population genetic expectations (Lovell et al., 2013, 2014, 2017). Lovell et al. (2017) used apomictic and sexual populations of *B. spatifolia* and investigated patterns of nucleotide variation across both reproductive modes through whole-genome sequencing. They found an elevated sequence diversity and heterozygosity, together with an increased mutation accumulation, in apomictic populations (Lovell et al., 2017). Likewise, in a larger survey of 37 natural populations of four *Boechera* spp. (*B. stricta*, *B. retrofracta*, *B. polyantha*, and *B. pendulocarpa)*, microsatellite markers showed the same trend (Lovell et al., 2013): higher levels of heterozygosity were found in apomicts compared to sexuals, independent of the ploidy level of the apomict.

In apomictic lineages, evolution occurs due to both genetic drift and natural selection (Charlesworth and Wright, 2001; Glémin et al., 2006; Brukhin and Baskar, 2019). The lower efficiency of selection expected in apomictic lineages would lead to an increased extinction risk of apomicts through the accumulation of deleterious alleles and an incapacity to adapt to environmental changes (Darlington, 1958; Muller, 1964; Bengtsson, 2009; Brukhin and Baskar, 2019). Contrary to these expectations, the genus *Boechera* is highly diverse, including several apomictic lineages (Alexander et al., 2013). A likely explanation for the survival of apomictic *Boechera* lineages is intra- and interspecific gene flow within the genus (Böcher, 1951; Sharbel and Mitchell-Olds, 2001; Dobeš et al., 2004a,b; Schranz et al., 2005; Beck et al., 2012; Lovell et al., 2013, 2017; Schilling et al., 2018). Gene flow mainly occurs from sexuals to apomicts, while apomicts are able to produce reduced pollen that can pollinate sexual lineages and transfer the dominant factor(s) conferring apomixis. Mutual gene flow between apomictic and sexual lineages may allow introgression of adaptive alleles from sexual into apomictic lineages, as posited by Van Dijk et al. (2009) in *Taraxacum* spp. Moreover, apomixis in *Boechera* spp. is facultative where different individuals may produce both sexual and apomictic offspring (Schranz et al., 2005; Aliyu et al., 2010). These probable instances of sexuality in apomicts may suffice to purge deleterious mutations and restore the fitness of apomictic lineages, securing their evolutionary survival (Van Dijk et al., 2009).

Differences in the strength of natural selection acting on sexual versus apomictic *B. spatifolia* populations were described by Lovell et al. (2014). The authors analyzed selection gradients by correlating genotypic trait means with relative fitness measurements, and found a reduction in the strength of adaptive evolution in apomictic relative to sexual lineages. Apomictic lineages experienced relatively less quantitative and molecular genetic differences between populations than sexuals. Also, divergence between apomictic populations was not correlated with environmental variation but, conversely, genomic structure and quantitative traits of sexual lineages were highly correlated with latitude, climatic variables, and elevation (Lovell et al., 2014). A common garden experiment revealed that flowering time was under strong selection in high-altitude sites. This is in agreement with studies in *B. stricta* (Anderson et al., 2011), which showed flowering time to be under directional selection in a study using recombinant inbred lines subjected to lab and field experiments.

Several studies assessed genetic dynamics and the extent of natural selection in *Boechera* populations. Earlier work on the population dynamics of the sexual species *B. fecunda* (Song and Mitchell-Olds, 2007) and *B. stricta* (Song et al., 2009), using sequence data from several nuclear loci and microsatellites, revealed similar levels of polymorphism and population differentiation in both species, regardless of the marked difference between the widespread *B. stricta* and the endangered *B. fecunda* with a reduced range. Similarly, studies comparing the widespread species *B. stricta* and *B. latifolia* with the rare species *B. crandallii* and *B. vivariensis* did not find strong associations between species size range and within-population genetic diversity (Lovell and McKay, 2015). However, the more widespread species exhibited higher phenotypic plasticity and quantitative trait structure (Qst), while the rare species contained stronger signatures of selection evidenced by higher Qst: Fst ratios, with Fst referring to the fixation index (Lovell and McKay, 2015). Extending the work of Song and Mitchell-Olds (2011) on *B. fecunda*, Leamy et al. (2014) found regional adaptation through extensive quantitative characterization of populations in Montana (United States) using microsatellite markers. Their analyses of genetic (Fst) and quantitative trait differentiation (Qst) showed evidence for divergent selection acting on water use efficiency and a contribution of the regional environmental conditions to local adaptation. Likewise, Lee and Mitchell-Olds (2011), using microsatellite markers and phenotypic quantitative analyses, demonstrated that water availability was the key environmental variable explaining genetic differentiation between two major genetic groups of *B. stricta* in Eastern and Western North America. All of these studies relied on microsatellite data and Fst estimations and should be interpreted with caution as microsatellite markers are not ideal for measuring population differentiation (Balloux and Lugon-Moulin, 2002; Putman and Carbone, 2014). Likewise, the use of Fst as a measure of population differentiation has been criticized (Jost, 2008; Meirmans and Hedrick, 2010; Whitlock, 2011; Jakobsson et al., 2013).

Whole-genome sequencing and chromosome painting on the same two major genetic groups of *B. stricta* investigated by Lee and Mitchell-Olds (2011) identified an inversion in Linkage Group 1 of the *B. stricta* genome (Lee et al., 2017). Populations carrying the inversion had lower polymorphism in Linkage Group 1, lower Tajima’s D, and more linkage disequilibrium than populations without the inversion. Furthermore, the inversion had a strong effect on flowering time in near-isogenic lines under greenhouse conditions. These results showed that this inversion has important ecological impacts on the species and that natural selection is driving the differentiation of *B. stricta* populations in North America (Lee et al., 2017).

Hybridization is common between members of the genus *Boechera* (Böcher, 1951; Sharbel and Mitchell-Olds, 2001; Dobeš et al., 2004a,b; Windham and Al-Shehbaz, 2007a,b). It was reported that hybridization occurs across the whole genus and happened repeatedly and independently (Schranz et al., 2005; Alexander et al., 2013). The earliest molecular evidence supporting hybridization comes from analysis of ITS and chloroplast sequence data, and gene flow between species now known as *B. stricta* and *B. retrofracta* was inferred by phylogeographic analyses (Dobeš et al., 2004a,b). It should be noted that conclusions on the hybrid nature of individuals, which are based on a single locus or an organellar genome, may not accurately reflect the history of a clade or population (Doyle, 1992; Maddison et al., 2006). However, Schranz et al. (2005) performed extensive crossing experiments, showing that successful crosses are possible among several members of the genus. This indicates a lack of intrinsic reproductive isolation barriers, and thus the possibility for extensive gene flow among different *Boechera* species.

Thus far, microsatellite markers have been central in identifying species and putative hybrids in the *Bochera* genus (Li et al., 2017). Beck et al. (2012) studied *Boechera* individuals using a set of 13 microsatellites. Hybrids between *B. fendleri* × *B. stricta* and *B. retrofracta* × *B. stricta* were confirmed using this methodology (Beck et al., 2012). Using similar methods, Lovell et al. (2013) studied 231 individuals from 37 natural populations of four *Boechera* species (*B. stricta, B. retrofracta, B. polyantha*, and *B. pendulocarpa*). They concluded that all triploid individuals were apomictic hybrids. This was not the case for diploid apomictic accessions, which behaved as true species rather than hybrid individuals. Based on these results, it was concluded that hybridization is an indirect correlate of apomixis in the genus *Boechera*.

With the advent of next generation sequencing technologies, the identification of hybrids is now more refined and precise. By using whole-genome sequencing in *B. spatifolia*, Lovell et al. (2017) investigated whether apomictic populations had a hybrid origin or not. Analysis of 22′000 haplotype trees across the genome indicated a hybrid origin of the apomictic *B. spatifolia* accessions. In another study using genotyping-by-sequencing methods, Schilling et al. (2018) assessed genomic variation in 79 individuals of eight *Boechera* species. Admixture analyses allowed to precisely identify hybrid individuals. This study provided evidence of recent and ancient admixture and variation across species.

## Inheritance and Genetic Aspects of Apomixis in the Genus *Boechera*

The seminal work of Nogler in the 1970es had shown that apomixis is genetically controlled (summarized in Nogler, 1984a). Subsequent crossing experiments of apomictic individuals as pollen donors with sexual maternal plants showed that apomixis is inherited as a dominant trait in many species (Grossniklaus et al., 2001). Early studies had indicated that apomixis is inherited as a single dominant locus, for instance in *Ranunculus auricomus* and *Panicum maximum*, where apomeiosis and parthenogenesis were found to cosegregate (Savidan, 1982; Nogler, 1984b). However, later studies found that different loci control the developmental components of apomixis, i.e., apomeiosis, parthenogenesis, and formation of functional endosperm, in most apomicts. It was also found that the genomic regions conferring apomixis or apomeiosis exhibit suppressed recombination (reviewed in Grossniklaus, 2001; Grossniklaus et al., 2001; Bicknell and Koltunow, 2004; van Dijk and Vijverberg, 2005; Barcaccia and Albertini, 2013; Hand and Koltunow, 2014; Hand et al., 2015; Brukhin, 2017). Apomixis is also frequently associated with hybridization and resulting polyploidy (Koltunow and Grossniklaus, 2003). The duplicated genomic load might be the cause of the deregulation, in space and time, of genes associated with sexual reproduction (Grimanelli et al., 2001; Grossniklaus, 2001; Spillane et al., 2001; Koltunow and Grossniklaus, 2003; Barcaccia and Albertini, 2013), as the newly formed polyploid hybrid faces the asynchronous expression of genes involved in reproduction (Carman, 1997, 2007; Grimanelli et al., 2001; Grossniklaus, 2001; Van Dijk, 2009). Apomixis, as an escape from sterility, has been speculated to be a transitional period in the evolution of neopolyploids, especially when facultative (Hörandl and Hojsgaard, 2012; Hojsgaard et al., 2014). Recent data indicate that apomixis is associated with increased diversity (Hojsgaard et al., 2014), suggesting that apomixis may actually contribute to the establishment of new polyploids (Hojsgaard, 2018) and to the diversification of angiosperms (reviewed in Brukhin and Baskar, 2019).

One of the unique features of apomixis in the genus *Boechera* is that it can occur at the diploid level. Diploid *Boechera* apomicts are highly heterozygous hybrids (Beck et al., 2012), and recent cytogenetic and population studies of the sexual and apomictic *Boechera* spp. have shown that these diploid genomes can be complex. Based on marker and ploidy analysis in diverse *Boechera* species, the emerging model proposes that, first, genetic factors for apomeiosis would independently arise. Such an individual, apomeiotic in the female side only, would stably generate seeds with a 2C embryo and 5C endosperm by self-pollination with reduced pollen. Other individuals might be apomeiotic on the male side only, generating seeds with a variety of ploidies. Reduced pollen from female-apomeiotic individuals would allow crossing with sexual individuals, thereby disseminating the phenotype. Over time, eventually both female- and male-apomeiotic individuals would cross, and the resulting seeds with a 2C embryo and 6C endosperm would become stable diploids with unreduced male and female gametes. These diploid apomicts, as they also produce fertile unreduced pollen, are then capable of fertilizing sexual diploids, which could result in triploid apomicts (Lovell et al., 2013).

Metaphase chromosome painting by genomic *in situ* hybridization demonstrated that all investigated apomictic lineages showed signs of a hybridogenic origin. All were found to be alloploid with a varying number of chromosomes inherited from either *B. holboellii s. l.* or *B. stricta.* The structure of their chromosomes was strongly affected by the consequences of hybridization, resulting in aneuploidy, and the replacement of homeologous chromosomes (Kantama et al., 2007). Therefore, these apomictic *Boechera* spp. are not univocal diploids, rather they have a polyhaploid origin (Sokolov et al., 2011). It should be noted that these cytogenetic data do not exclude the possible existence of true diploids among *Boechera* spp., although they cast doubt on it. Inheritance of apomixis-related traits has been proposed to be associated with the heterochromatic chromosomes *Het*, *Het’*, and *Del* found in apomictic diploids (Kantama et al., 2007). According to Kantama et al. (2007), all diploid apomictic accessions examined had at least four *B. stricta* chromosomes, including *Het* and *Del*, and the combination of these chromosomes might be important for the manifestation of apomixis.

Recent studies have shown that the *Het* chromosome is the altered homolog of the first chromosome of *B. stricta*, which underwent an accumulation of pericentromeric heterochromatin, while the *Het’ + Del* pair is the result of *Het* breakage followed by a pericentric inversion in the *Het’* chromosome (Mandáková et al., 2015). According to earlier data, in some lineages *Del* could have resulted from a translocation fusing the proximal segment of the *B. stricta* chromosome to the distal segment of the *B. holboellii s. l.* chromosome (Kantama et al., 2007). However, hybridizing sexual and apomictic *Boechera* accessions failed to produce apomictic progeny, despite the inheritance of the *Het* chromosome (Schranz et al., 2006). When crossing sexual *B. stricta* diploids with apomictic *B. divaricarpa* allodiploids carrying the *Het* chromosome, the F1 offspring were triploid and had low fertility but were not apomictic despite carrying the *Het* chromosome. The F2 population displayed an array of ploidy levels and chromosome numbers, and an equally low fertility. The few F3 individuals seemed to maintain the high ploidy of their parents and fertility increased relative to their F1 and F2 ancestors, but did not reach the levels of the individuals used in the original cross. In any case, there were no apomictic progeny produced. Thus, the genetic control of apomixis in *Boechera* spp. is not limited to the inheritance of aberrant chromosomes (Schranz et al., 2006).

Chromosomal regions with suppressed recombination around apomixis-related genes, often in a hemizygous state and enriched with repeat sequences and transposons, has been found in many phylogenetically distant apomicts, both dicots, and monocots (Grossniklaus et al., 2001; Ozias-Akins et al., 2003; Van Dijk et al., 2009; Okada et al., 2011; Kotani et al., 2014). It is assumed that such a recombinationally inert region can contain several linked genes with different functions, the synergistic effects of which could lead to apomictic development. However, in many apomicts, the loci controlling the different components of apomixis are in distinct regions of the genome. In *Boechera* spp., the most likely candidates to carry such loci are the aberrant chromosomes *Het, Het’*, and *Del*. Taking into account the hybridogenic nature of *Boechera* apomicts as a mechanism that triggered the emergence and subsequent evolution of such recombinationally inert blocks bearing apomixis-related genes, hybridization of species with incomplete chromosomal homology may have resulted in the formation of non-recombinant, hemizygous regions from which such blocks evolved (Sharbel et al., 2010).

Several theories speculate on the mechanisms that control apomixis. Gene mutations have the appeal of the master regulator hypothesis, in which the mutation of a gene upstream of a regulatory cascade would lead to apomeiosis, parthenogenesis, and/or autonomous endosperm development (Koltunow and Grossniklaus, 2003), or the acummulation of mutations in low-recombining regions for each aspect of apomixis, as evidenced in some of the aforementioned apomictic species. There have been various mutants identified in *Arabidopsis* that lead to apomeiotic phenotypes (Schmidt et al., 2015). Interestingly, they seem divided between cell-cycle regulators/core meiotic genes (Ravi et al., 2008; d’Erfurth et al., 2009, 2010; Zhao et al., 2017), and genes involved in small RNA (sRNA) pathways (Olmedo-Monfil et al., 2010; Schmidt et al., 2011).

While no mutants have yet been studied in *Boechera*, two loci have been identified which correlate with female and male apomeiosis. The *APOmixis Linked Locus* (*APOLLO*), which encodes an Asp-Glu-Asp-Asp-His exonuclease, is down-regulated in sexual ovules when they enter meiosis and up-regulated in apomeiotic ovules (Corral et al., 2013). *APOLLO* shows biallelic inheritance with “apo-” and “sex-” alleles. These alleles differ in a 20-nucleotide polymorphism in the 5′ untranslated region of the exonuclease gene. All tested apomictic *Boechera* accessions were heterozygous for the *APOLLO* alleles, having at least one apoallele and one sexallele, while all sexual genotypes are homozygous for sexalleles (Corral et al., 2013). *APOLLO*’s male counterpart is the *Unreduced Pollen GRAin Development2* (*UPGRADE2*) locus, which is exclusively expressed in PMCs of apomictic species. It encodes a chimeric long non-coding RNA (lncRNA) with the potential to form stable secondary structures. *UPGRADE2* arose from duplication of *UPGRADE1*, followed by insertion of a functional gene and subsequent exonization, which made it transcriptionally active (Mau et al., 2013). There is a high correlation between the presence of these apomixis-associated loci and the apomictic mode of reproduction (98.4% for *APOLLO*, 96% for *UPGRADE2* in 275 *Boechera* accessions from 22 species), although it was also found that, in sexuals, 2.27% had the *APOLLO* apoallele and 34.48% *UPGRADE2*. Although *APOLLO* is thus the most suitable diagnostic indicator of apomixis in different *Boechera* species and accessions (Mau et al., 2015), its function during reproduction has not yet been elucidated. The independence of *APOLLO* and *UPGRADE2* is consistent with population genetic studies, which showed that male and female apomeiosis are inherited independently, although they usually correlate with each other at the population level (Lovell et al., 2013).

Kliver et al. (2018) found two additional, more distant copies of *APOLLO*, which may indicate past duplication events. An examination of apo- and sex-alleles of *APOLLO* indicates that they arose after the separation of the *Boechera* genus and form two separate clades. Given that *B. retrofracta* and *B. stricta* are sexual species, it was not surprising that they carried sex-alleles of *APOLLO* (Kliver et al., 2018). The authors suggest an evolutionary scenario where, after triplication that likely took place before the separation of the Brassicaceae, one of the *APOLLO* copies might have acquired a novel function in the common ancestor of *Boechera* spp., leading to the separation of the apomictic lineages. The Ka/Ks ratio of *APOLLO* alleles indicates that the branch leading to the apo-alleles is under positive selection (Ka/Ks = 1.4646), which is typical for paralogs that acquired a novel function.

Epigenetic changes in gene regulation have also been proposed to lie at the origin of apomixis. It has been demonstrated that in *A. thaliana* polyploidization following interspecific hybridization leads to dramatic changes in gene expression (Lee and Chen, 2001), making it a suitable unifier of both the hybridization and gene mutation hypothesis, whereby epialleles rather than mutant alleles would play an initial role in deregulating reproductive genes in space and time (Grimanelli et al., 2001; Grossniklaus, 2001; Spillane et al., 2001; Koltunow and Grossniklaus, 2003). sRNAs have been implicated in epigenetic reprogramming during gametogenesis and post-fertilization events (Martinez and Köhler, 2017), and of the genes involved in the sRNA pathway, *AGO9* has been shown to interact with 24-nucleotide sRNAs derived from transposable elements in ovules. It is not clear if the apomeiotic phenotype of ago9 mutants is due to the lack of silencing transposable elements, or a consequence of other sRNAs that interact with AGO9 (Vielle-Calzada et al., 2012). In *Boechera*, sRNA expression profiling revealed *Boechera*-specific conserved sRNAs and microsatellite-like RNAs (misRNA), many of which have potential binding sites in exonic regions, the majority of their targets being regulatory factors. The quantitative variation in misRNA target binding was hypothesized to result from microsatellite-length polymorphisms either in their precursors or target genes, which could account for transcriptome-wide shifts in gene regulation between sexuals and apomicts (Amiteye et al., 2013). Such a shift has, in fact, been observed not only in apomictic versus sexual *Boechera*, but the apomictic ovule has also a significant overrepresentation of transcription factors activity (Sharbel et al., 2010; Schmidt et al., 2014), as well as a significantly different regulation of core cell cycle, sRNA pathway genes (Schmidt et al., 2014), and heterochronic differences in imprinted genes (Sharbel et al., 2010).

Although the genes controlling the components of apomixis in *Boechera* spp. are not yet identified, the data on the inheritance of apomixis and on apomixis-associated loci provide valuable entry points for further studies. Ultimately, functional studies of candidate genes will be required and the experimental tools required for such analysis need to be further developed.

## Molecular Tools for the Genus *Boechera*: Transformation, Laser Capture Microdissection, and Transcriptomics

One of the most useful tools to study molecular mechanisms is transformation for stable transgene expression. *Agrobacterium*-mediated transformation has been the method of choice whenever possible, as any DNA sequence contained between the two tumor-inducing (Ti) borders of the plasmid can efficiently be introduced into a plant genome (Gelvin, 2003). While the “floral dip” method is standard for stable transformation of *A. thaliana* (Clough and Bent, 1998), most plant species require more elaborate transformation procedures. The most widely used method is co-cultivation of explants with *Agrobacterium*, which then transform into callus tissue and subsequently undergo organogenesis to regenerate a transformant plant. With this method, even recalcitrant cultivars of various crops can be successfully transformed (reviewed in Altpeter et al., 2016). Several sexual and apomictic *Boecher*a species have been investigated for their potential to be transformed by *Agrobacterium*, and it was reported to be possible to regenerate shoots from hypocotyl-derived calli of sexual *B. stricta* and apomictic *B. gunnisoniana* and *B. holboellii* (Taşkin et al., 2003, 2015). Somatic embryos derived from immature cotyledons of the apomict *B. divaricarpa* (Taşkin et al., 2009b) and, as a proof-of-concept, stable transformants of *B. gunnisoniana* were also generated (Taşkin et al., 2003). These advances open up the genus *Boechera* to the possibilities offered by the study of transgenic lines.

Major advances have also been made in the characterization of transcriptomes in sexual and apomicitic *Boechera* spp. Microarrays were first used to describe transcriptomes in *Boechera* spp., followed by various sequence-based approaches, including SuperSAGE using Sanger sequencing (Matsumura et al., 2006). Currently, RNA-sequencing (RNA-seq), based on next generation sequencing technologies, is the method of choice to study transcriptomes (Wang et al., 2009). Continuous methodological improvements now allow high precision and high throughput studies of single cell (Picelli et al., 2014), live (Lovatt et al., 2014), and low input (Schmidt et al., 2012; Florez-Rueda et al., 2016) transcriptomes. Not surprisingly, most transcriptomic studies on *Boechera* spp. focused on differences in expression between apomictic and sexual accessions (Sharbel et al., 2009, 2010; Amiteye et al., 2011, 2013; Aliyu et al., 2013; Schmidt et al., 2014; Shah et al., 2016), while a minority focused on the ecological interactions of plants with their environment (Cano et al., 2013; Gill et al., 2016; Kannan et al., 2018).

The first transcriptomic studies in *Boechera* spp. were performed by Sharbel et al. (2009, 2010) using the SuperSAGE technique (Matsumura et al., 2006). They quantified gene expression in manually dissected ovules at the MMC stage of two sexual (*B. stricta* and *B. holboelli*) and two apomictic accessions (both *B. divaricarpa*). Additionally, two cDNA libraries representing apomictic and sexual accessions were sequenced using Roche’s 454 technology (Sharbel et al., 2009). These were the first reference transcriptomes for the genus *Boechera* and formed the basis for future studies (see below). In a second study, Sharbel et al. (2010) quantified gene expression between a single apomict and a single sexual *Boechera* individual at four different developmental time points but without biological or technical replication. Stage-specific and heterochronic patterns of gene expression were identified (Sharbel et al., 2010). Because these first transcriptomic studies (Sharbel et al., 2009, 2010) used single libraries from single individuals without biological replication, they cannot account for variation between individuals and lack the statistical power for a robust identification of genes that are differentially expressed in sexuals versus apomicts (Lee et al., 2000;Meyers, 2004; Conesa et al., 2016).

The sRNA fraction of the *Boechera* transcriptome (Amiteye et al., 2011) was identified by a reanalysis of transcriptome data (Sharbel et al., 2009) and the sequencing of two sRNA libraries (Amiteye et al., 2013). Using a *Boechera*-specific microarray based on the sexual and apomictic reference transcriptomes (Sharbel et al., 2009), an analysis of copy number variation (CNV) in transcriptionally active regions of 10 sexual and 10 apomictic *Boechera* accessions was performed (Aliyu et al., 2013). The gene ontology classes found enriched in apomictic CNVs (e.g., pollen-pistil interaction), led to the hypothesis that CNV in these gene classes serves to buffer the effects of deleterious mutations.

The first attempt to compare sexual and apomictic development in *Boechera* spp. at the cellular level was pioneered by Schmidt et al. (2014). While previous studies used whole ovules (Sharbel et al., 2009, 2010), they used laser-assisted microdissection (LAM) to isolate the apomictic initial cell (AIC), nucellus, egg, central, and synergid cells of the triploid apomict *B. gunnisoniana* (Schmidt et al., 2014). After LAM, cDNA libraries were produced and sequenced using SOLiD technology. A reference transcriptome from pooled floral tissues of *B. gunnisoniana* was sequenced with Illumina technology (Schmidt et al., 2014). An analyses of gene expression and gene ontology enrichment uncovered the upregulation of spermidine metabolism and patterns of altered expression in the AIC. Comparison to female gametophyte cell-specific transcriptomes of *A. thaliana* (Wuest et al., 2010) identified regulatory pathways that differ between sexual and apomictic germlines, including hormonal, epigenetic, cell cycle control, and transcriptional regulatory pathways (Schmidt et al., 2014). Likewise, comparison of egg cell-specific transcriptomes of *B. gunnisoniana* and *A. thaliana* identified genes expressed only in the apomictic egg cell (Florez-Rueda et al., 2016). Future studies that exploit single-cell transcriptomics by comparing apomictic and sexual *Boechera* spp. are expected to shed light onto the molecular basis of apomixis in the genus *Boechera*.

A study of apomictic and sexual *Boechera* seedlings focused on the response to abiotic and biotic conditions and stress-specific changes that might underlie apomixis (Shah et al., 2016). A relationship between apomixis and environmental conditions is also supported by a phenomenon known as “geographical parthenogenesis” (reviewed in Hörandl, 2006). This concept is based on the observation that apomictic lineages have larger distributional ranges than their sexual relatives. In *Boechera* spp., niche differentiation was found to be driven by ploidy rather than reproductive mode (Mau et al., 2015), indicating low support for geographical parthenogenesis in the genus. Nevertheless, the great variation in ploidy level and reproductive mode and its relationship with niche differentiation make the genus *Boechera* a good model for the study of plant-environment interactions (Rushworth et al., 2011). From this perspective, the transcriptomes of an obligate triploid apomict and a diploid sexual, both isolated from a drought-prone habitat, were compared. Specific meiotic genes were found to be down-regulated and stress-related transcription factors and chaperons upregulated in apomictic seedlings (Shah et al., 2016), but the relevance of these findings for reproduction is unknown.

## Genomic Resources for the Genus *Boechera* and Challenges to Genome Analysis

The advent of next generation sequencing along with progress in bioinformatics tools opened a new chapter in the study of apomixis, allowing the search for apomixis-associated loci and the comparison, genotyping, and phylogenetic analysis of *Boechera* species and accessions using whole-genome sequencing.

Currently, the genomes of only two *Boechera* spp. have been assembled and published (Table 2). Both *B. stricta* and *B. retrofracta* are self-pollinating, diploid sexuals and have largely homozygous genomes, which are straightforward to assemble. Notably, repeats in the genome of *B. retrofracta* occupy almost 40% of the genome space. Nearly half of them are long terminal repeats (LTRs) (18.27%) (Kliver et al., 2018). In contrast, only 20% of the *B. stricta* genome are annotated as repeats (Lee et al., 2017, assembly v1.2). The difference in the repeats number correlates with the difference in their genome sizes of the (Table 2). In some apomictic species, the apomixis loci are associated with heterochromatin and/or substantial repetitive sequences (Hand and Koltunow, 2014). The chromosomes carrying the *LOSS-OF-APOMEIOSIS* (*LOA*) locus in *Hieracium praelatum* and the *APOSPORY-SPECIFIC GENOMIC REGION* (*ASGR*) in *Pennisetum squamulatum* are characterized by extensive repetitive sequences and transposon-rich regions (Okada et al., 2011). In apomictic *Paspalum simplex*, the region containing apomixis-related loci has undergone large-scale rearrangements due to transposable elements (Calderini et al., 2006). These similarities in repetitive, heterochromatic regions in the genomes of apomicts have led to the hypothesis that these regions might serve as a sink to sequester factors involved in sexual reproduction, triggering apomixis (Grossniklaus, 2001; Koltunow and Grossniklaus, 2003). In line with this idea, some *Boechera* apomicts have largely heterochromatic chromosomes (Kantama et al., 2007) and some transposon families were found enriched in an apomictic *Boechera* lineage (Aliyu et al., 2013). Due to extensive hybridization within the *Boechera* genus, however, the repeat content of the genome might not reflect the mode of reproduction but rather its phylogeographic history.

**Table 2:** Publicly available genome assemblies of *Boechera* spp.

	Assembly	N50,	Genes	
Species, accession	size, Mb	Mb	annotated	BUSCO benchmarks^1^	Reference/access URL
**Sexual diploid *Boechera spp.***					
*B. stricta*, LTM	189.34	2.18	27′416	C:97.4%(S:89.2%, D:8.2%),	Lee et al., 2017
				F:1.3%, M:1.3%, *n*:1440	https://www.ncbi.nlm.nih.gov/genome/?term=txid72658[orgn]
					https://phytozome.jgi.doe.gov/pz/portal.html#!info?alias=Org_Bstricta
*B. retrofracta (formerly B. holboellii), Panther*	222.25	2.29	27′048	C:95.2%(S:87.1%, D:8.1%)	Kliver et al., 2018
				F:0.6%, M:4.2%, *n*:1440	http://hdl.handle.net/11701/15405
					http://public.gen-watch.org/ad89dedc8b4674276c9b0760f29b07af/


*^*1*^Annotated proteins were used for BUSCO benchmarking C, complete; S, complete-single-copy; D, complete-duplicated; F, fragmented; M, missing BUSCO groups were found (Waterhouse et al., 2017).*

The final genome annotation of *B. stricta* and *B. retrofracta* encompassed about 27′000 genes in both species. The presence of a slightly greater number of predicted transcripts in *B. stricta* can be explained by lack of gene expression data for *B. retrofracta*, which resulted in a less complete gene annotation overall, as confirmed by BUSCO benchmarking (Table 2).

**FIGURE 5:**
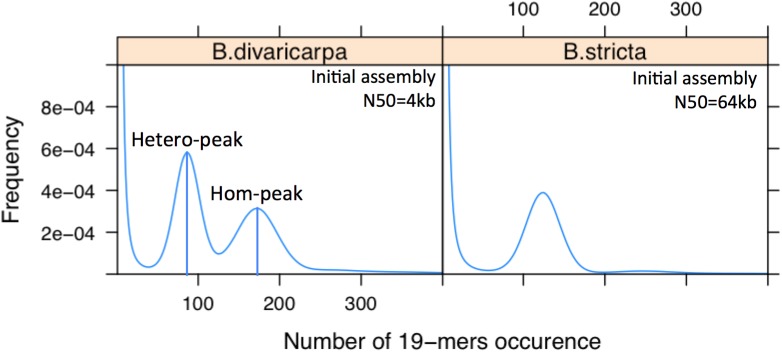
The *B. divaricarpa* genome is extremely heterozygous. Distribution of the number of 19-mer occurrences in *B. stricta*, which has only one peak, indicating a highly homozygous genome, and *B. divaricarpa*, which has 2 peaks with a pronounced difference in height. Note that the number of occurrences of different K-mers in the first peak (ca. 90) is half of that in the second peak (ca. 180), suggesting that first and second peak represent the heterozygous and homozygous part of the genome, respectively. N50 values are based on the assembly of Illumina paired-end reads (100× coverage) by Platanus. Heterozygosity has an immediate effect on the contiguity of the genome assembly.

Assembling the genomes of diploid apomictic *Boechera* species is difficult because they exhibit high levels of heterozygosity (Figure 5), which results from the combination of disparate genomes as consequence of their hybridogenic origin (Beck et al., 2012). For example, the genome heterozygosity rate of *B. divaricarpa* is around 2.5% as estimated by GenomeScope (Vurture et al., 2017). Because of all the reasons mentioned above, sequencing and *de novo* assembly of such a plant genome can result in a highly fragmented genome draft. Annotation of the protein coding genes may not always be correct, considering that nearly identical genes are notoriously difficult to assemble. Thus, a mosaic sequence can be formed that does not represent any member of the gene family. The high level of fragmentation and mis-assembly could prevent our ability to draw true conclusions about the evolution of apomixis-associated loci and the molecular mechanisms underlying this interesting phenomenon (Claros et al., 2012).

A key challenge is the assembly of the short reads into contiguous sequences (contigs), which then are assembled into chromosome-scale scaffolds. Another complication is the assignment of genetic variants to the correct homeologous chromosome, a process known as haplotyping (Korbel and Lee, 2013).

Only recently, approaches have been developed that are capable to solve the problem of heterozygous genome assembly. Pacific Biosciences long-read sequencing technology and FALCON/FALCON-Unzip algorithms were used to assemble heterozygous genomes including an F1 hybrid of *A. thaliana* and the widely cultivated *Vitis vinifera* cv. Cabernet Sauvignon (Chin et al., 2016). Further development of this assembler resulted in FALCON-Phase, a new method that reconstructs contig-length phase blocks using Hi-C short-reads, which is able to produce true diploid assemblies (Kronenberg et al., 2018). Linked-Read sequencing technology (10× Genomics) has recently been successfully employed for a *de novo* assembly of the heterozygous F1 diploid pepper (*Capsicum annuum*) hybrid genome (Hulse-Kemp et al., 2018).

Recently, methods for a haplotype-aware, phased assembly of polyploid genomes were developed for cases where the parental species are known (Akama et al., 2014; Kyriakidou et al., 2018). However, speciation in the genus *Boechera* has a very complex history, where sexual diploids gave rise to multiple apomictic species through hybridization-associated polyploidy, alloploidy, and aneuploidy. This complexity of speciation resulted in the unprecedented genome diversity of the apomictic species in this genus, which was further exaggerated by mutation accumulation (Lovell et al., 2017) and elevated transposon activity in the apomicts (Ferreira de Carvalho et al., 2016). It is thus often not clear what the parental ancestors of polyploid *Boechera* spp. are. Genomic analysis based on a haploid reference genome might not reflect the reality, especially for apomicts with highly heterozygous diploid/polyploid genomes. In such a situation, many loci might be completely absent in the reference genome because even in the ideal case, it represents only a consensus genome. Bearing in mind also the problems in producing genome assemblies for apomictic species, we would like to outline the potential of an alternative, reference-free approach for comparative genomic analyses of sexual and apomictic species in the genus *Boechera*.

A reference-free (or, more general, alignment-free) approach to sequence comparison does not rely on alignment and, therefore, it is especially valuable for analyzing genomes of organisms that do not have a reference (Zielezinski et al., 2017). K-mer or word frequency method is one of the most popular alignment-free method for comparative genome analysis, but its successful application can be hindered by insufficient sequencing depth and biases of genome sampling. Illumina paired-end (PE) sequencing of random-primed libraries produce the most suitable data for processing by this approach. Table 3 provides a selection of next generation sequencing data from *Boechera* spp. that satisfy the requirements for alignment-free methods.

**Table 3:** Publicly available genomic sequencing data^1^ of *Boechera* genus suitable for reference-free (k-mer based) analysis.

	Dataset
Species, accession	size^2^	SRA#	Reference
**Sexual diploid *Boechera* species**			
*B. stricta*, LTM	425×	SRR396760	Lee et al., 2017
		SRR396762	
		SRR396756	
*B. stricta*, SAD12	225×	SRR1592624	–
*B. retrofracta (formerly B. holboellii), Panther*	420×	SRR3929707	Kliver et al., 2018.
*B. arcuata, San Diego 0097*	285×	SRR6448790	–
*B. spatifolia, Rosita3*	48×	SRR5116719	Lovell et al., 2017
*B. spatifolia, Tiesiding2*	32×	SRR5116723	Lovell et al., 2017
*B. spatifolia, Cripple6*	38×	SRR5116724	Lovell et al., 2017
*B. spatifolia, Chiquito7*	29×	SRR5116726	Lovell et al., 2017
*B. spatifolia, Alvarado2_1*	12×	SRR5116728	Lovell et al., 2017
*B. spatifolia, Alvarado1_3*	18×	SRR5116729	Lovell et al., 2017
*B. spatifolia, Royal2*	36×	SRR5116730	Lovell et al., 2017
*B. spatifolia, Chicago2*	30×	SRR5116732	Lovell et al., 2017
**Apomictic** **diploid** ***Boechera*** **species**			
*B. divaricarpa*, ES517	750×	SRR3500627	–
		SRR3500628	
*B. perennas, San Diego 193153*	377×	SRR6448882	–
*B. spatifolia, Tiesiding7*	31×	SRR5116718	Lovell et al., 2017
*B. spatifolia, Rosita4*	33×	SRR5116720	Lovell et al., 2017
*B. spatifolia, Royal1*	32×	SRR5116721	Lovell et al., 2017
*B. spatifolia, Chiquito4*	44×	SRR5116722	Lovell et al., 2017
*B. spatifolia, Chicago4*	37×	SRR5116725	Lovell et al., 2017
*B. spatifolia, Cripple7*	36×	SRR5116727	Lovell et al., 2017
*B. spatifolia, Alvarado1_2*	29×	SRR5116731	Lovell et al., 2017
*B. spatifolia, Alvarado2_2*	28×	SRR5116733	Lovell et al., 2017
**Apomictic polyploid *Boechera* species**			
*B. depauperata^3^, Yosemite 224299*	317×	SRR6448869	–


*^*1*^Illumina pair-end libraries only. ^*2*^Times of typical *Boechera* genome size (220Mbases). ^*3*^Reproductive mode assignment is based on k-mer profile.*

**FIGURE 6:**
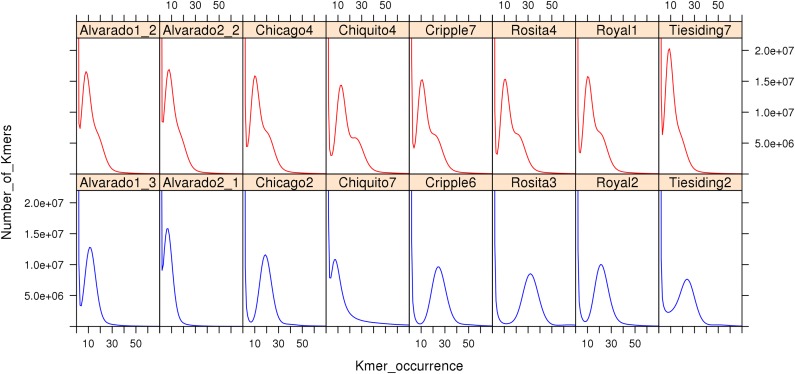
K-mer (27) profiles of genomic sequence reads (Illumina) from apomictic (red) and sexual (blue) *B. spatifolia*. Note characteristic shape of the right slope on the graphs in all apomictic accessions.

To compare sexual and apomictic accessions using the K-mer method, we reanalyzed *B. spatifolia* sequencing data (Lovell et al., 2017). Figure 6 shows K-mer (*K* = 27) profiles of sequencing reads from the eight sympatric pairs of sexual and apomictic *B. spatifolia* genotypes (Lovell et al., 2017). The K-mer profile provides an estimate of effective sequence coverage and reflects the rate of genome heterozygosity, the amount of sequencing errors along with errors of sample preparation and sequencing data processing (Supplementary Note 1 in Vurture et al., 2017). As seen in Figure 6, profiles of apomictic individuals are clearly distinguishable from profiles of sexual individuals, even for a K-mer coverage as low as 10. The K-mer profiles of the apomictic individuals is shaped by a higher level of heterozygosity compared to sexual individuals (Li et al., 2017) and, in case of high sequence coverage, the profile contains two peaks which represent the heterozygous and homozygous part of the genome.

**FIGURE 7:**
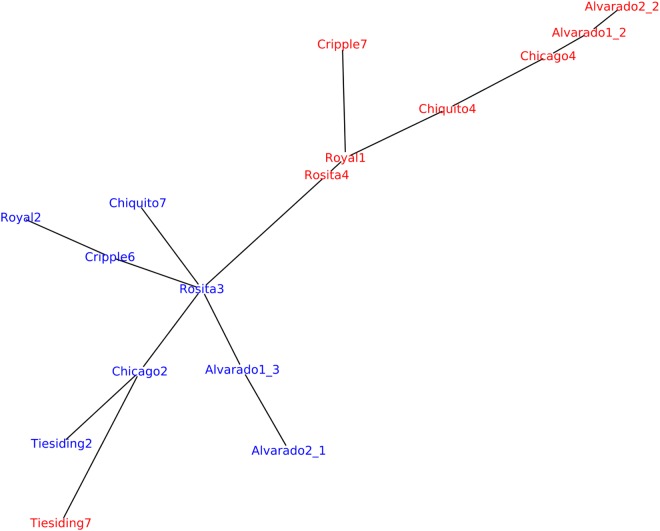
Replicating the analysis of *B.* s*patioflia* genetic structure variation (Lovell et al., 2017). Sequencing reads from 16 re-sequenced individuals (8 apomict-sexual sympatric pairs) were used to generate a minimum spanning network where edge length is proportional to Mash distance, which estimates the mutation rate between genomic sequences of *B. spatiofolia* individuals directly from their MinHash sketches (Ondov et al., 2016). Nodes represent sexual (blue) and apomictic (red) individuals.

We also used an alignment-free method to investigate genetic relatedness in the *B. spatiofolia* individuals analyzed by Lovell et al. (2017). Figure 7 shows genetic variation analysis analogous to the one described by Lovell et al. (2017, Figure 1B) but using Mash software for genetic distance estimation (Ondov et al., 2016). In contrast to Lovell et al. (2017) who used *A. lyrata* as reference for the alignment of *B. spatiofolia* sequencing reads, this approach is based solely on the data contained in the reads. Nevertheless, the resulting tree is rather similar.

## Conclusion

Apomixis produces progeny that is genetically identical to the mother plant, a trait of great agronomical importance. Unfortunately, the molecular mechanisms underlying apomixis are only poorly understood. A better understanding of the genetic networks that control the components of apomixis are crucial for its introduction into crop plants. Apomicts of the genus *Boechera* represent a convenient model to study apomixis as it also occurs at the diploid level and genomes of *Boechera* spp. are comparatively small. Despite these advantages, genome assembly and annotation of the apomictic *Boechera* lineages is complicated due to such phenomena as a high level of heterozygosity of their genomes, which results from chromosome rearrangements, accompanied by alloploidy, aneuploidy, and substitutions of homeologous chromosomes occurring during hybridization events. The use of next generation sequencing and novel bioinformatic approaches should help to overcome these challenges and facilitate generating the first comprehensive genome of an apomictic plant in the near future. Attempts to apply reference-free methods for the assembly and comparative analysis of such genomes are currently underway. A combination of systems biology approaches to analyze RNAseq and genomic data from sexual and apomictic *Boechera* species, as well as functional approaches in transgenic plants, will facilitate the disentanglement of the genetic control of apomixis at the molecular level. This is prerequisite for the engineering self-sustaining, apomictic hybrids in sexual crop plants.

## Data Availability

All datasets generated for this study are included in the manuscript and/or the supplementary files.

## Author Contributions

VB and UG designed and directed the study, wrote the introduction and conclusion, and advantages of the Boechera genus for the study of apomixis. JO and VB analyzed the systematic position of Boechera and habitats. JO, MSN, and VB carried out cyto-embryological studies in the Boechera genus. AF-R and UG worked on population genetics of Boechera with respect to apomixis. VB and MSN performed the inheritance and genetic aspects of apomixis in Boechera. MSN and AF-R performed the molecular experiments in Boechera: transformation, laser capture microdissection, and transcriptomics. DS and EB analyzed the available NGS data and genomic resources for Boechera. EB inquired the transcriptomic investigations for analysis of apomictic plants. All authors read and approved the final manuscript.

## Conflict of Interest Statement

The authors declare that the research was conducted in the absence of any commercial or financial relationships that could be construed as a potential conflict of interest. The reviewer AJ declared a past co-authorship with one of the authors UG to the handling Editor.

## Footnotes

1 https://reeis.usda.gov/web/crisprojectpages/1000552-cytological-and-molecular-characterizations-of-reproduction-in-sexual-and-apomictic-boechera-brassicaceae.html
